# Development and characterization of a guinea pig model for Marburg virus

**DOI:** 10.24272/j.issn.2095-8137.2017.054

**Published:** 2018-02-09

**Authors:** Gary Wong, Wen-Guang Cao, Shi-Hua He, Zi-Rui Zhang, Wen-Jun Zhu, Estella Moffat, Hideki Ebihara, Carissa Embury-Hyatt, Xiang-Guo Qiu

**Affiliations:** 1 Special Pathogens Program, National Microbiology Laboratory, Public Health Agency of Canada, Winnipeg, Manitoba R3E 3R2, Canada; 2 Shenzhen Key Laboratory of Pathogen and Immunity, State Key Discipline of Infectious Disease, Shenzhen Third People’s Hospital, Shenzhen Guangzhou 518020, China; 3 Key Laboratory of Pathogenic Microbiology and Immunology, Institute of Microbiology, Chinese Academy of Sciences, Beijing 100101, China; 4 Department of Medical Microbiology and Infectious Diseases, University of Manitoba, Winnipeg, Manitoba R3E 0J9, Canada; 5 National Center for Foreign Animal Disease, Canadian Food Inspection Agency, Winnipeg R3E 3M4, Canada; 6 Department of Molecular Medicine, Mayo Clinic, Rochester, Minnesota 55905, USA

**Keywords:** Marburg virus, Guinea pig, Animal model, Pathogenesis, Host adaptation

## Abstract

The Angolan strain of Marburg virus (MARV/Ang) can cause lethal disease in humans with a case fatality rate of up to 90%, but infection of immunocompetent rodents do not result in any observable symptoms. Our previous work includes the development and characterization of a MARV/Ang variant that can cause lethal disease in mice (MARV/Ang-MA), with the aim of using this tool to screen for promising prophylactic and therapeutic candidates. An intermediate animal model is needed to confirm any findings from mice studies before testing in the gold-standard non-human primate (NHP) model. In this study, we serially passaged the clinical isolate of MARV/Ang in the livers and spleens of guinea pigs until a variant emerged that causes 100% lethality in guinea pigs (MARV/Ang-GA). Animals infected with MARV/Ang-GA showed signs of filovirus infection including lymphocytopenia, thrombocytopenia, and high viremia leading to spread to major organs, including the liver, spleen, lungs, and kidneys. The MARV/Ang-GA guinea pigs died between 7–9 days after infection, and the LD_50_ was calculated to be 1.1×10^–1^ TCID_50_ (median tissue culture infective dose). Mutations in MARV/Ang-GA were identified and compared to sequences of known rodent-adapted MARV/Ang variants, which may benefit future studies characterizing important host adaptation sites in the MARV/Ang viral genome.

## INTRODUCTION

Marburg virus (MARV) is a pathogen belonging to the family *Filoviridae*, genus *Marburgvirus*, and is classified as a Risk Group 4 Pathogen (Phac-Aspc.Gc.Ca, 2011). MARV is considered to be extremely pathogenic in humans. Initial symptoms of infection are nonspecific with high grade fever, chills, headache and muscle aches followed by nausea, vomiting, diarrhea and abdominal pain. Progression of disease to advanced stages is characterized by the appearance of maculopapular rash, increased vascular permeability, neurological signs, severe dehydration, diffuse coagulopathy, hemorrhage, shock and multi-organ failure leading to death within 7–10 days after symptoms appear (Mehedi et al., 2011). The few patients that survive the infection enter a prolonged convalescence phase but are beset with complications including ocular symptoms, joint pain, orchitis and psychosis, among others (Mehedi et al., 2011). Additionally, MARV is known to persist in some survivors and can be spread via semen (Brainard et al., 2016), potentially resulting in secondary cases of infection. There are currently no specific preventatives or cures approved for treating MARV infections. 

The first MARV outbreak was reported during 1967 in Marburg, Germany, in which 7 deaths from 31 total infections were recorded. The source of the virus was eventually traced to infected monkeys imported from Uganda (Kissling et al., 1968; Smith et al., 1967). A total of 12 outbreaks have been recorded so far, with the last occurring in Uganda during 2014 (CDC Gov, 2014). The largest and deadliest MARV outbreak occurred during 2004–2005 in Angola when 227 fatal cases out of 252 infections were reported, for a case fatality rate (CFR) of ~90% (Towner et al., 2006). Experimental infection of cynomolgus macaques with the Angolan strain of MARV (MARV/Ang) suggests that this virus appears to cause a more rapid onset and severe outcome of infection, compared to other MARV strains (Fernando et al., 2015). Live MARV has been isolated from bats (Towner et al., 2009) and several outbreaks have been associated through contact with wild bats inside mines and caves (Adjemian et al., 2011; CDC, 2009; Timen et al., 2009), strongly suggesting that bats play a major role in transmission of the virus to humans.

Due to their low unit costs and ease of handling, rodents are particularly desirable for initial studies *in vivo*, especially for the screening of candidate antivirals against infectious diseases. However, immunocompetent rodents are not susceptible to infection with wild-type MARV isolates. To address this issue, we had previously developed a variant of MARV/Ang via serial passaging in the livers of severe combined immunodeficiency (SCID) mice (Qiu et al., 2014). This novel, mouse-adapted variant (MARV/Ang-MA) caused uniformly lethal disease when injected intraperitoneally (i.p.) into BALB/c and C56BL/6 mice, replicating to high levels in the blood, intestines, kidneys, lungs, brain, spleen, and livers. Other findings include lymphocytopenia, thrombocytopenia and marked damage to the liver and spleen (Qiu et al., 2014). The MARV/Ang-MA model is currently being used to assess the potential of candidate drugs against MARV (Warfield et al., 2017).

Non-human primates (NHPs) are widely considered to be the best animal model available for recapitulating human MARV disease, and any candidate antivirals must show efficacy in NHPs before clinical trials. However, it is prudent to develop and characterize an alternate small animal model to confirm any findings from mice before testing in NHPs, as these animals are costly, laborious to handle, and require specialized housing. To address this need, we developed a MARV/Ang variant adapted to guinea pigs (MARV/Ang-GA) by sequential passaging in the livers and spleens of these animals. MARV/Ang-GA was then characterized with regards to median lethal dose (LD_50_), viremia/biodistribution at various times after infection. The full length viral genome of MARV/Ang-GA was also sequenced to identify any mutations arising from the passaging process, and compared to the wild-type MARV/Ang as well as available sequences of rodent-adapted MARV/Ang to elucidate potential “hotspots” for adaptation mutations.

## MATERIALS AND METHODS

### Cells and viruses

The wild-type, clinical virus isolate Marburg virus/H.sapiens-tc/AGO/2005/Angola, or MARV/Ang, was isolated from a patient during the 2005 Marburg outbreak in Uige, Angola. The adapted virus is called Marburg virus/NML/C.porcellus-lab/AGO/2005/Ang-GA-P2, or MARV/Ang-GA. Stocks of MARV/Ang and MARV/Ang-GA were grown in T-150 flasks (Corning, USA) of Vero E6 cells (ATCC, USA) for these studies.

### Animals and virus passaging

Outbred female, 4–6 week old strain Hartley guinea pigs between 200–250 g in weight were used for passaging experiments. Two guinea pigs were injected i.p. with ~10^6^ plaque forming units (pfu) of MARV/Ang in 1 mL of Dulbecco's modified Eagle's medium (DMEM), supplemented with 2% heat-inactivated fetal bovine serum (FBS) (Sigma, Canada). Serial passaging of the virus was then performed as follows. Livers and spleens were removed from euthanized animals at 7 days post infection (dpi), pooled, and homogenized by grinding the livers against a steel mesh with a plastic plunger. Splenocytes and hepatocytes were suspended in 10 mL of phosphate-buffered saline (PBS) and centrifuged at 400 r/min for 5 min. The supernatant was passed through a 70 µm cell strainer, and the cell pellet was mechanically homogenized in 1 mL DMEM with sterile steel beads in a tissue homogenizer. After centrifugation at 400 r/min for 5 min, the supernatant from this cell pellet was also passed through the 70 µm cell strainer. Two naïve guinea pigs were injected i.p. with 1 mL of the homogenate, and the process was repeated again at 7 dpi until MARV/Ang-GA was produced after 9 passages. MARV/Ang-GA was not plaque purified. 

All animal work was approved by the Animal Care Committee (ACC) at the Canadian Science Center for Human and Animal Health (CSCHAH), and performed in accordance with the guidelines from the Canadian Council on Animal Care (CCAC). Studies involving live, infectious virus were performed under biosafety level 4 (BSL-4) conditions at the National Microbiology Laboratory located in Winnipeg, Canada.

### LD_50_ determination

Serial 10-fold dilutions of MARV/Ang-GA from 2.32×10^−2^–2.32×10^2^ TCID_50_ (median tissue culture infective dose) was administered per guinea pig (*n*=3 per group). Animals were weighed daily and monitored for survival and weight loss. 

### Serial sampling of infected guinea pigs

Guinea pigs were infected with 1 000×LD_50_ of MARV/Ang-GA, or an equivalent dose of MARV/Ang. Four animals from each group were euthanized at 0, 3, 5 and 7 dpi for necropsy. Blood was sampled from anesthetized guinea pigs by cardiac puncture. Whole blood was collected in K2 EDTA plus blood collection tubes (BD Biosciences, Canada) for blood counts and stored in lysis buffer AVL (Qiagen, USA) for determination of viremia, and lithium heparin blood collection tubes (BD Biosciences, Canada) for blood biochemistry. Major organs including livers, spleen, kidneys and lungs were harvested from these animals at the same timepoints and stored in RNAlater for subsequent analysis.

### Blood counts and blood biochemistry

Complete blood counts and blood biochemistry analyses were performed with a VetScan HM5 hematology system or a VetScan VS2 analyzer, respectively (Abaxis Veterinary Diagnostics, USA).

### Determination of viral titers by reverse transcription-quantitative PCR (RT-qPCR)

RNA was extracted from whole blood using the QIAamp viral RNA minikit (Qiagen, USA) or from tissue homogenates with an RNeasy minikit (Qiagen, USA), following manufacturer instructions. The amount of viral genome was quantified via RT-qPCR on the ABI StepOnePlus, using the LightCycler 480 RNA master hydrolysis probe kit (Roche, Canada) with the RNA polymerase as the target gene. Reaction conditions were as follows: 63 °C for 3 min, 95 °C for 30 s, and then 45 cycles of 95 °C for 15 s and 60 °C for 30 s. Primer and probe sequences were as follows: forward, 5′-GCAAAAGCATTCCCTAGTAACATGA-3′; reverse, 5′-CACCCCTCACTATRGCGTTYTC-3′; probe, 6-carboxyfluorescein [FAM]-TGGCACCAYAATTCAGCAAGCAT AGG-black hole quencher[BHQ].

### Histopathology and immunohistochemistry

Tissues were fixed in 10% neutral phosphate buffered formalin, routinely processed, sectioned at 5 µm and stained with hematoxylin and eosin (HE) for histopathologic examination. For immunohistochemistry (IHC), paraffin tissue sections were quenched for 10 min in aqueous 3% hydrogen peroxide. Epitopes were retrieved using an in-house glyca retrieval solution, in a Biocare Medical Decloaking Chamber. The primary antibody applied to the sections was an in-house anti-MARV mouse monoclonal antibody (3H1). It was used at a 1:4 000 dilution for 30 min. They were then visualized using a horse radish peroxidase labelled polymer, Envision®+system (anti-mouse) (Dako, USA) and reacted with the chromogen diaminobenzidine (DAB). The sections were then counter stained with Gill’s hematoxylin.

### Statistical analysis

Statistical analysis was performed with the two-way ANOVA with Bonferroni post-tests. Statistical significance was set at *: *P*<0.05, **: *P*<0.01, or ***: *P*<0.001.

### Sequencing of MARV/Ang-GA

RNA was extracted from MARV/Ang and MARV/Ang-GA stocks using a Qiagen QIAmp viral RNA minikit (Qiagen, USA), following manufacturer instructions. Sanger sequencing of MARV/Ang-GA is carried out using the same protocol as described in a previous publication (Qiu et al., 2014).

### Nucleotide sequence accession number

The sequence of MARV/Ang-GA was deposited in GenBank (Accession No. MF939097).

## RESULTS

### LD_50_ determination of MARV/Ang-GA in guinea pigs

**Figure 1:**
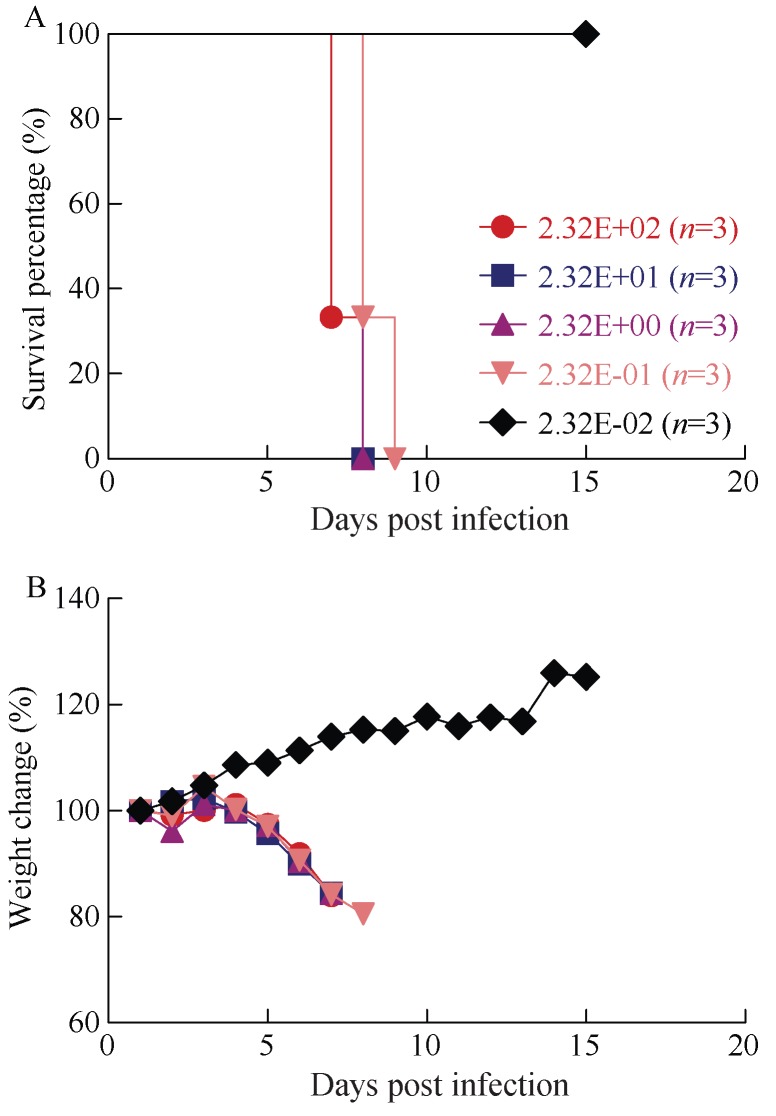
LD_50_ determination of MARV/Ang-GA in guinea pigs

Groups of three guinea pigs were inoculated i.p. with serial 10-fold dilutions of MARV/Ang-GA, ranging from 2.32×10^–2^–2.32×10^2^ TCID_50_, in order to assess the LD_50_ of this adapted virus. Survival and weight loss was monitored for 15 days after challenge. The animals that were challenged with 2.32×10^0^–2.32×10^2^ TCID_50_ of MARV/Ang-GA all succumbed to disease on 7–8 dpi (the mean time to death for the 2.32×10^2^ TCID_50_ group was 7.3±0.6 dpi) with close to 20% weight loss (Figures 1A, B). The animals that were challenged with 2.32×10^–1^ TCID_50_ of MARV/Ang-GA died on 8–9 dpi with almost 20% weight loss (Figures 1A, B). The animals given 2.32×10^–2^ TCID_50_ of MARV/Ang-GA all survived the challenge with no weight loss observed (Figures 1A, B). Using the linear regression from the logarithmic value of the challenge titer and the fatality rate, the LD_50_ was estimated to be approximately 1.1×10^–1^ TCID_50_.

A–B: Survival (A) and weight loss (B) of guinea pigs infected intraperitoneally with serial 10-fold dilutions of MARV/Ang-GA ranging from 2.32×10^–2^–2.32×10^2^ TCID_50_ per animal.

### Clinical overview of changes in guinea pigs after infection with MARV/Ang or MARV/Ang-GA

**Figure 2:**
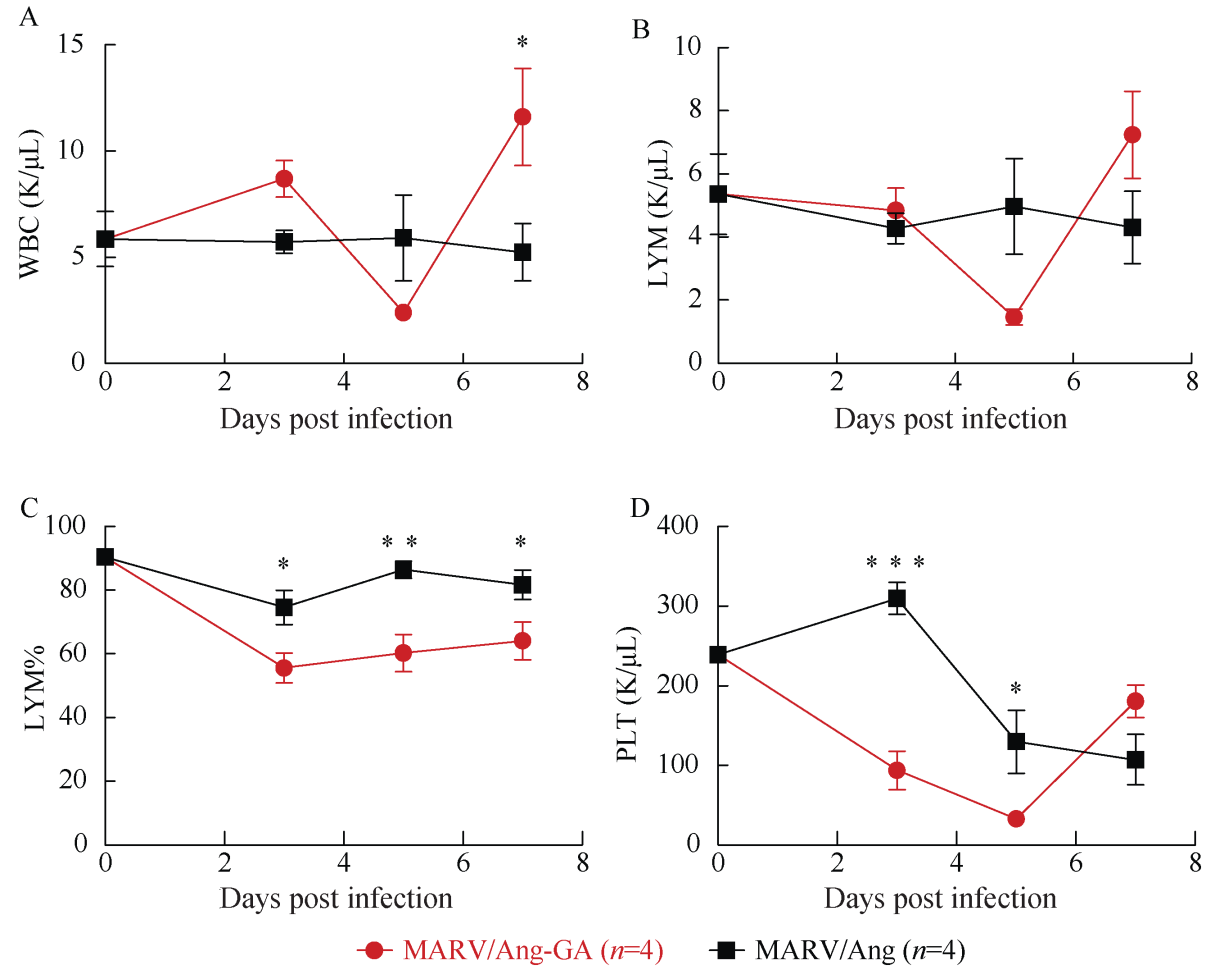
Complete blood counts of guinea pigs challenged with 1 000×LD_50_ of either MARV/Ang-GA or MARV/Ang

**Figure 3:**
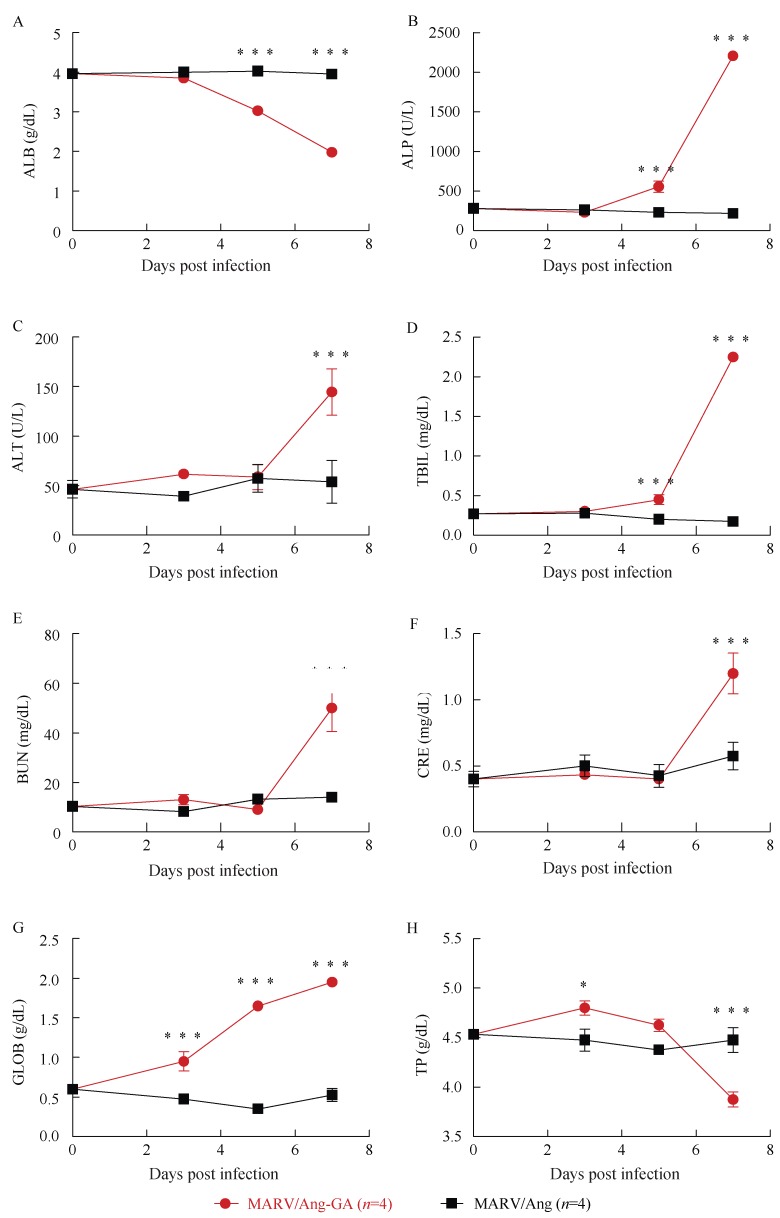
Blood biochemistry of guinea pigs challenged with 1 000×LD_50_ of either MARV/Ang-GA or MARV/Ang

Groups of three guinea pigs were then challenged i.p. with 1 000×LD_50_ (or 110 TCID_50_) of MARV/Ang-GA, or an equivalent dose of MARV/Ang. As expected, weight loss and clinical symptoms were only observed in the MARV/Ang-GA animals, but not in the MARV/Ang group (Supplementary Figure 1A, B). A complete blood count as well as biochemical analysis was performed from whole blood drawn from infected animals at 0, 3, 5 and 7 dpi. A drop in the counts of white blood cells and lymphocytes was observed in MARV/Ang-GA animals at 5 dpi (Figures 2A, B), whereas the decrease in lymphocyte percentage was apparent and statistically significant from 3–7 dpi (Figure 2C). Additionally, a significant decrease in platelet count was observed in MARV/Ang-GA animals at 3–5 dpi, whereas this decrease was more gradual and less pronounced in MARV/Ang animals (Figure 2D). Advanced to terminal disease in MARV/Ang-GA guinea pigs can be observed by statistically significant increases in the following markers: albumin, alkaline phosphatase, alanine aminotransferase, total bilirubin, blood urea nitrogen, creatinine and globulin, while these markers stayed relatively constant in MARV/Ang animals (Figures 3A–G). Additionally, a decrease in total protein levels was observed in MARV/Ang-GA guinea pigs at 7 dpi, but not in MARV/Ang animals (Figure 3H).

A: White blood cells (WBC); B: Lymphocytes (LYM); C: Lymphocyte percentage (LYM%); D: Platelet (PLT); *: *P*<0.05; **: *P*<0.01; ***: *P*< 0.001.

A: Albumin (ALB); B: Alkaline phosphatase (ALP); C: Alanine aminotransferase (ALT); D: Total bilirubin (TBIL); E: Blood urea nitrogen (BUN); F Creatinine (CRE); G: Globulin (GLOB); H: Total protein (TP); *: *P*<0.05; **: *P*<0.01; ***: *P*<0.001.

### Viremia and biodistribution of MARV/Ang or MARV/Ang-GA in guinea pigs

**Figure 4:**
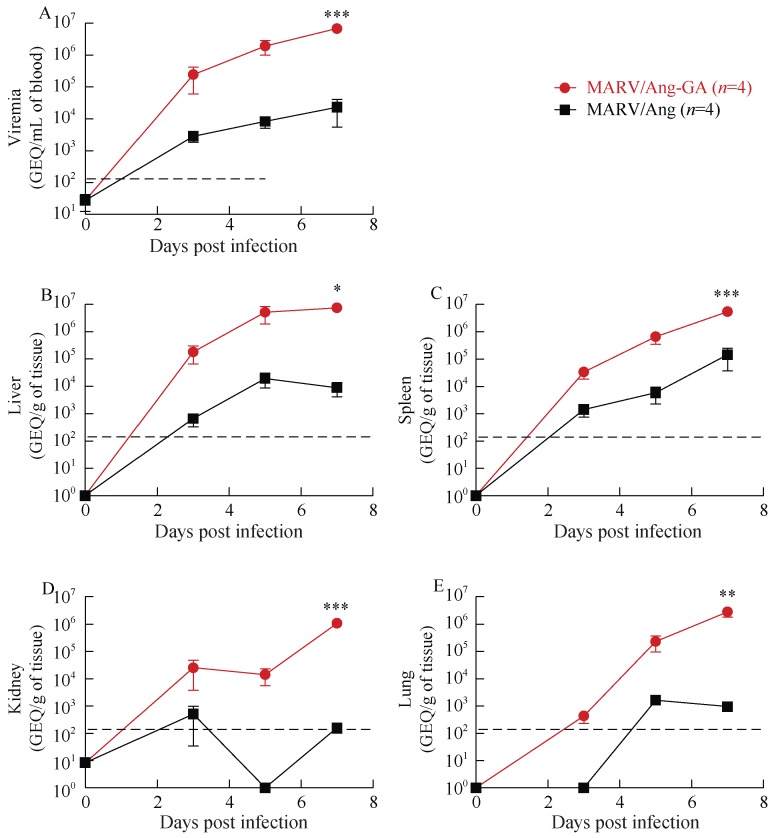
Viremia and viral biodistribution of guinea pigs challenged with 1 000×LD_50_ of either MARV/Ang-GA or MARV/Ang

The amount of MARV/Ang or MARV/Ang-GA in the blood and selected major organs of infected guinea pigs were quantified by RT-qPCR at 0, 3, 5 and 7 dpi. Animals infected with MARV/Ang showed evidence of viral replication, with viremia of ~10^3^ rising to ~10^4^ genome equivalents per mL of blood (GEQ/mL blood) from 3–7 dpi, whereas those infected with MARV/Ang-GA showed much higher levels of replication, with ~10^5^ rising to 10^7^ GEQ/mL blood from 3–7 dpi (Figure 4A). MARV/Ang was found at substantial levels in the livers and spleens of guinea pigs, with peak values of ~10^4^ and ~10^5^ GEQ per gram of tissue (GEQ/g tissue), respectively, whereas for MARV/Ang-GA the peak values in the liver and spleen were ~10^7^ GEQ/g tissue (Figures 4B, C). While substantial levels of MARV/Ang-GA was found in the kidneys and lungs of infected animals with peak values of ~10^6^ GEQ/g tissue in both organs at 7 dpi, MARV/Ang is not as readily detectable in the kidneys or lungs, with peak values of ~10^3^ GEQ/g tissue for both organs (Figures 4D, E).

The amount of viral RNA was quantified by RT-qPCR in the blood (A), liver (B), spleen (C), kidney (D) and lungs (E) of the infected animals; the dashed lines represent the limit of detection with the RT-qPCR assay; *: *P*<0.05; **: *P*<0.01; ***: *P*<0.001.

### Histopathology and immunohistochemistry

**Figure 5:**
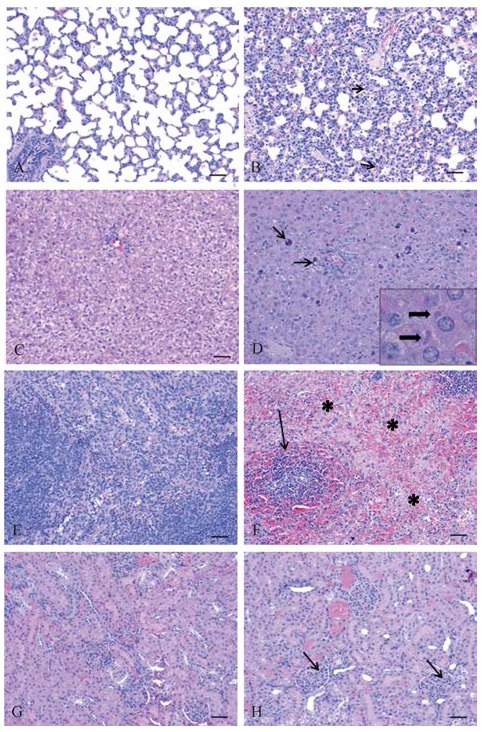
Histopathology findings in uninfected versus GA-MARV infected guinea pigs

**Figure 6:**
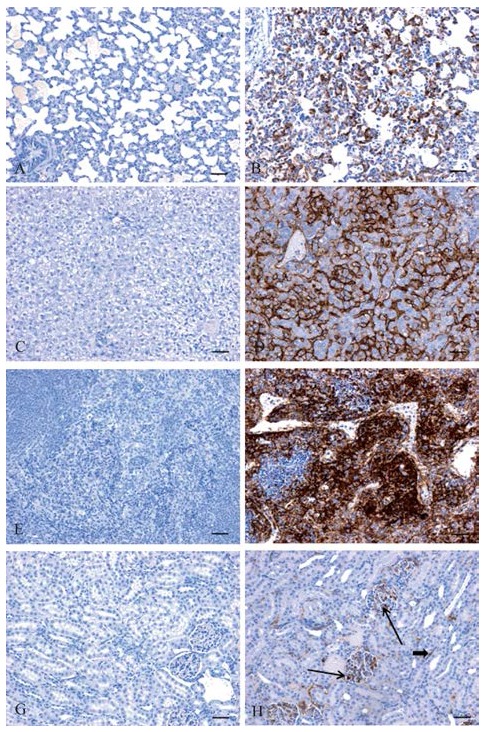
Immunohistochemistry findings in uninfected versus GA-MARV infected guinea pigs

In histopathology results, lesions were observed in all tissues examined from infected animals but no lesions were observed in uninfected control animals (Figures 5A–H). In the lungs the alveolar walls were expanded by infiltration of inflammatory cells and there was extensive individual cell necrosis. Throughout the liver there was degeneration of hepatocytes often with mineralization as well as portal inflammation and intracytoplasmic inclusion bodies. In the spleen there was massive necrosis of cells within both the red and white pulp areas. Mild lesions in the kidneys were characterized by scattered individual degeneration of cells within glomeruli. In immunohistochemistry results viral antigen was detected in all infected animals including lung alveolar walls, sinusoids/ hepatocytes of the liver, red and white pulp of the spleen and within the renal glomeruli however no immunostaining was observed in uninfected control animals (Figures 6A–H).

Lung (A), liver (C), spleen (E) and kidney (G) from uninfected control guinea pigs were normal and no significant lesions were detected. In infected guinea pigs lesions were observed in all tissues; B: In the lung, the alveolar walls contained numerous inflammatory cells and there were many degenerating/necrotic cells throughout (arrows); D: Liver lesions included hepatocyte degeneration/necrosis often with mineralization (thin arrows) and numerous intracytoplasmic viral inclusion bodies were observed (inset); F: In the spleen, there was massive necrosis in both the red pulp (*) and white pulp (arrow) areas; H: In the kidneys, there were degenerating cells with pyknotic nuclei scattered throughout the glomeruli (arrows). Scale bars: 20 µm.

No positive immunostaining for MARV viral antigen was observed in lung (A), liver (C), spleen (E) or kidney (G) from uninfected control guinea pig. Abundant MARV antigen was detected in lung (B), liver (D), spleen (F) and kidney (H, arrows point to cells infected within glomeruli) from infected guinea pigs. Scale bars: 20 µm.

## Mutations in MARV/Ang-GA

The adapted virus MARV/Ang-GA was then sequenced and aligned with the publicly available sequence of MARV/Ang (GenBank accession No. DQ447653), in order to identify any mutations that arose and became fixed through passaging. A total of eight mutations were identified: two mutations in non-coding regions (base pairs 2 931 and 18 713), two mutations in the polymerase (L) coding region (base pairs 13 115 and 17 249) resulting in silent mutations at the amino acid level, one mutation in the viral protein 40 (VP40) coding region (base pair 4 735) resulting in a N56K change at the amino acid level, and three mutations in the viral protein 24 (VP24) coding region (base pairs 10 402, 10 853 and 10 931) resulting in changes of V66I, L216S and N242S, respectively, at the amino acid level (Table 1).

**Table 1:** Summary of mutations in MARV/Ang-GA, compared to MARV/Ang

Genome position (bp)	Coding (gene) or non-coding	MARV/Ang	MARV/Ang-GA Cross et al., 2015	MARV/Ang-GA (this study)	Amino acid change?
2 931	Non-coding	U	A	A	N/A
4 735	VP40	U	A	A	N56K
10 402	VP24	G	A	A	V66I
10 853	VP24	U	C	C	L216S
10 931	VP24	A	A	G	N242S
13 115	L	U	C	C	Silent mutation
17 249	L	U	A	A	Silent mutation
18 713	Non-coding	C	A	A	N/A
19 105	Non-coding	A	U	A	N/A

N/A: not applicable.

## DISCUSSION

Using the well-established method of passaging wild-type filovirus isolates in the livers and spleens of rodents, we successfully generated and characterized a guinea pig model against MARV/Ang, currently the most lethal strain of MARV to humans. Animals infected with MARV/Ang-GA developed severe disease characterized by weight loss, decrease in white blood cells and platelets, as well as increased levels/activities of markers for renal, hepatic and pancreatic functions, indicating multi-organ failure. High viremia and systemic spread of the virus was detected during the course of the disease.

Another research group had also reported on the development of a guinea pig animal model based on MARV/Ang (Cross et al., 2015). In their model, animals infected with 5 000 pfu of the guinea pig-adapted virus experience fever and weight loss resulting in death between 8–10 dpi. Gross pathology, histopathological and immunohistochemical studies show massive hepatic and splenic damage. The hematology and biochemical findings show lymphocytopenia and thrombocytopenia, but marked increases in liver-associated enzyme and pro-inflammatory cytokine levels. Additionally, they also show that guinea pigs infected with the adapted virus have coagulopathy, as evidenced by increased prothrombin and activated partial thromboplastin time, as well as decreased thrombin times and protein C activity (Cross et al., 2015).

Since the virus passaging by Cross et al. (2015) was performed completely independently from our study, this provided a rare opportunity to compare viral genome sequences between their guinea pig-adapted virus versus our MARV/Ang-GA. The purpose was to see if any common mutations exist between the two adapted viruses, which may give indications of the relative importance of each mutation. To our surprise, the two viruses were remarkably similar. All mutations in the coding and non-coding regions were identical, save for the N242S mutation in VP24 described in our study that was not present in their variant, and a mutation in a non-coding region (base pair 19 105) that was described in their study but undetected in our viral stock (Table 1). This suggested that the common mutations between our two viral stocks were the critical changes needed to attain virulence in guinea pig; however, it should be noted that some of the mutations may have been introduced during the preparation of the virus stock in VeroE6 cells, as previously demonstrated with a mouse-adapted MARV/Ang (Wei et al., 2017). It will be interesting to sequence the virus from the last *in vivo* passage from the guinea pigs and compare with the sequence of our viral stock. Additionally, in-depth studies using the reverse genetics system for MARV will be needed to elucidate the impact of each mutation.

Another important finding is that although non-coding regions and silent mutations are not expected to theoretically have an impact on virus evolution, the similarity of the mutations in these locations between the two viruses, despite independent passaging, suggests that the non-coding regions and silent mutations may play a larger-than-expected role. It will be important to also sequence the viral homogenates during each passage to observe when the mutations described arise and become fixed, and whether the mutations always appear in the same order. These studies will help determine whether viral evolution and host adaptation is based on a set template plan, and may give important clues on predicting viral evolution and host adaptation during subsequent MARV outbreaks.
